# Exploring three-dimensional orbital imaging with energy-dependent photoemission tomography

**DOI:** 10.1038/ncomms9287

**Published:** 2015-10-05

**Authors:** S. Weiß, D. Lüftner, T. Ules, E. M. Reinisch, H. Kaser, A. Gottwald, M. Richter, S. Soubatch, G. Koller, M. G. Ramsey, F. S. Tautz, P. Puschnig

**Affiliations:** 1 Peter Grünberg Institut (PGI-3), Forschungszentrum Jülich, 52425 Jülich, Germany; 2 Jülich Aachen Research Alliance (JARA), Fundamentals of Future Information Technology, 52425 Jülich, Germany; 3 Institute of Physics, University of Graz, NAWI Graz, Universitätsplatz 5, 8010 Graz, Austria; 4 Physikalisch-Technische Bundesanstalt (PTB), Abbestraße 2-12, 10587 Berlin, Germany

## Abstract

Recently, it has been shown that experimental data from angle-resolved photoemission spectroscopy on oriented molecular films can be utilized to retrieve real-space images of molecular orbitals in two dimensions. Here, we extend this orbital tomography technique by performing photoemission initial state scans as a function of photon energy on the example of the brickwall monolayer of 3,4,9,10-perylene tetracarboxylic dianhydride (PTCDA) on Ag(110). The overall dependence of the photocurrent on the photon energy can be well accounted for by assuming a plane wave for the final state. However, the experimental data, both for the highest occupied and the lowest unoccupied molecular orbital of PTCDA, exhibits an additional modulation attributed to final state scattering effects. Nevertheless, as these effects beyond a plane wave final state are comparably small, we are able, with extrapolations beyond the attainable photon energy range, to reconstruct three-dimensional images for both orbitals in agreement with calculations for the adsorbed molecule.

## Abstract (web summary)

Experimental data from angle-resolved photoemission spectroscopy can be utilized on molecular films to retrieve real-space images of molecular orbitals in two dimensions. Here, by scanning initial states as a function of photon energy, the authors can reconstruct three-dimensional orbital images.

## Graphical abstract

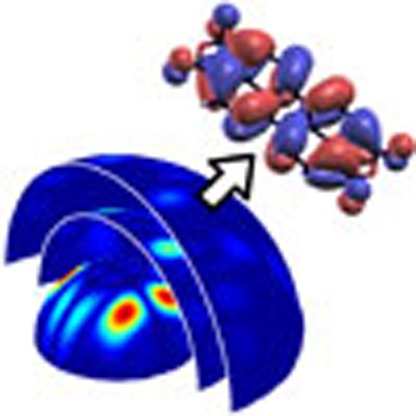

In recent years, a renaissance of angular-resolved photoemission spectroscopy (ARPES) in the field of organic electronics could be observed^1^,^2^,^3^,^4^,^5^,^6^,^7^,^8^,^9^,^10^,^11^,^12^,^13^,^14^,^15^,^16^,^17^,^18^,^19^,^20^,^21^,^22^,^23^,^24^,^25^,^26^,^27^. This development was mainly driven by the fact that, in opposition to conventional wisdom, the angular dependence of the photoemission current from oriented molecular films can be understood by assuming a plane wave as the final state of the photoemission process. This approximation enables a simple and intuitive interpretation of the transition matrix element in terms of the Fourier transform of the initial state orbital. Thereby a technique, called orbital tomography^8^,^11^,^14^,^23^, emerged which enables one to deconvolute photoemission spectra into individual orbital contributions. It provides an orbital-by-orbital characterization of large adsorbate systems allowing one to directly estimate the effects of bonding on individual orbitals and also yields most stringent tests for *ab initio* electronic structure theory^8^,^9^,^24^,^25^. The reciprocal relationship between the ARPES intensity and the spatial distribution of the initial orbital has also been utilized to study the hybridization of molecular states with the substrate^6^,^7^, explore intermolecular band dispersions^15^,^16^,^25^, shed light on the role of intermolecular versus molecular-substrate interactions^12^,^18^,^26^, determine molecular orientations^10^,^19^,^21^, reveal the nature of doping-induced states^20^, or even enable the reconstruction of molecular orbitals from ARPES data^5^,^17^,^22^. The success of experimentally obtaining two-dimensional (2D) orbital images including phase is encouraging to expand the technique to three dimensions. If possible, this would be extremely exciting, since presently experimental three-dimensional (3D) imaging of orbitals could only be demonstrated by femtosecond laser spectroscopy using high harmonics generation suitable only for simple diatomic molecules such as N_2_ in gas phase^28^.

Within the one-step model of photoemission and the plane wave final state approximation, the measured ARPES intensity is proportional to the square of the absolute value of Fourier transform 
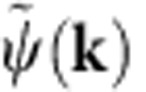
 of the initial orbital 
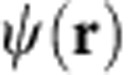
. This relationship between a molecular orbital in real space 
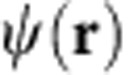
 and in momentum space 
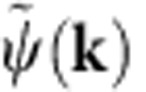
 is illustrated in Fig. 1 for the example of the lowest unoccupied molecular orbital (LUMO) of perylene-3,4,9,10-tetracarboxylic dianhydride (PTCDA) calculated from density functional theory (DFT). Panel (a) displays the LUMO orbital 
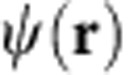
 in a conventional manner as a particular isosurface of the wave function, and panel (b) depicts its 3D Fourier transform 
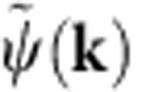
. Red and blue colours indicate particular isosurfaces at positive and negative values, respectively. For later reference, we have numbered four main lobes of the Fourier transform. Their positions in *k*-space determine the main spatial frequencies visible in the real space orbital. We have also plotted two Ewald-like spheres corresponding to kinetic energies of 29.7 eV (yellow, corresponding to a photon energy of 35.0 eV) and 58.1 eV (grey, photon energy 63.4 eV), respectively, which intersect these lobes and lead to the *k*_*x*_*k*_*y*_-momentum maps observable in constant binding energy (CBE) photoemission experiments with large angular acceptance^5^. Experimentally, such CBE maps of the PTCDA highest occupied molecular orbital (HOMO) and LUMO have already been measured for some selected photon energies of 21.2 and 30.0 eV, respectively, and used for the deconvolution of the experimental density of states^6^,^8^. Moreover, as we have shown in a recent publication, it is also possible to retrieve the phase information by means of an iterative, numerical procedure which only assumes the wave function to be confined to a spatial region determined by the van-der-Waals extension of the molecule. With this additional information on the phase (id est sign) of the momentum space wave function, it is possible to reconstruct the two-dimensional spatial distribution of the molecular orbital via an inverse Fourier transform^17^.

It is intriguing to ask whether it is also possible to recover the full three-dimensional spatial distribution of a molecular orbital from photoemission experiments. Judging from Fig. 1b, this appears possible, if the photoemission experiments were performed for a series of photon energies *hν*. With increasing *hν*, that is, accordingly increasing kinetic energy of emitted electrons for the fixed binding energy corresponding to the chosen molecular orbital, various hemispherical cuts through momentum space with a radius given by


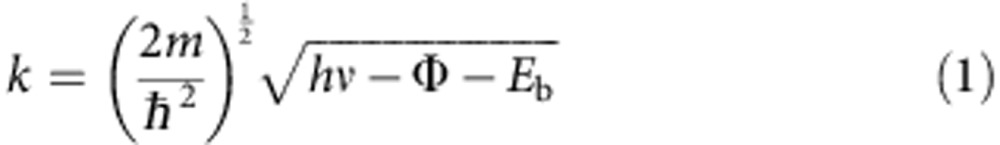


would eventually map out the complete 3D dependence of 
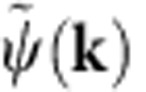
 on *k*_*x*_, *k*_*y*_ and *k*_*z*_. Note that in equation 1, expressing energy conservation, Φ denotes the work function of the PTCDA/Ag(110) brickwall monolayer of 4.5 eV and *E*_b_ the binding energy (with respect to the Fermi level) of the molecular state under investigation. In analogy to the 2D case, an inverse Fourier transform of the full 3D ARPES data should lead to the 3D orbital in real space.

In this work, we have done a carefully calibrated photon energy-dependent study of the HOMO and LUMO photoemission intensity. Using this data together with extrapolations, we show that the 3D reconstruction is possible despite small deviations from a plane wave final state being observed.

## Results

### Momentum maps

**Figure 1:**
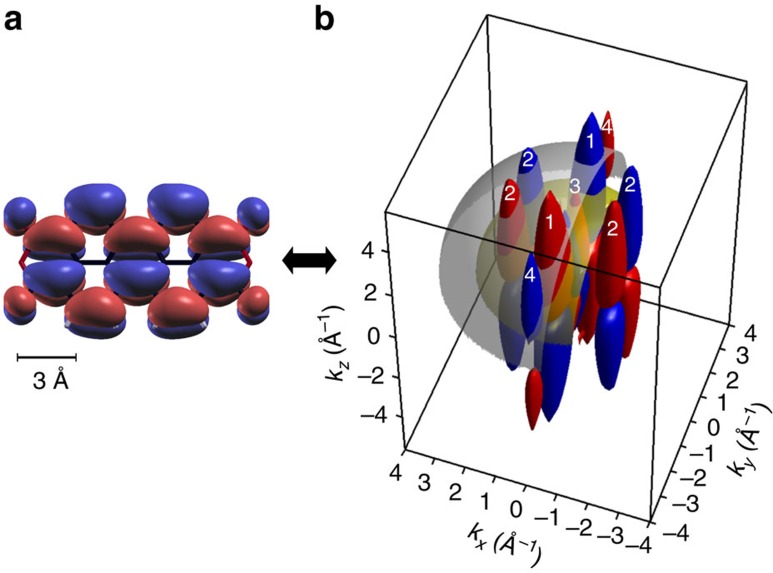
The LUMO of a free PTCDA molecule in real and momentum space. (**a**) and (**b**) Real and momentum space images, respectively, where the red (blue) color indicates isosurfaces of the wave function with positive (negative) sign. The four main lobes in momentum space are numbered, and hemispherical cuts corresponding to kinetic energies of 29.7 and 58.1 eV are indicated in yellow and grey, respectively.

**Figure 2:**
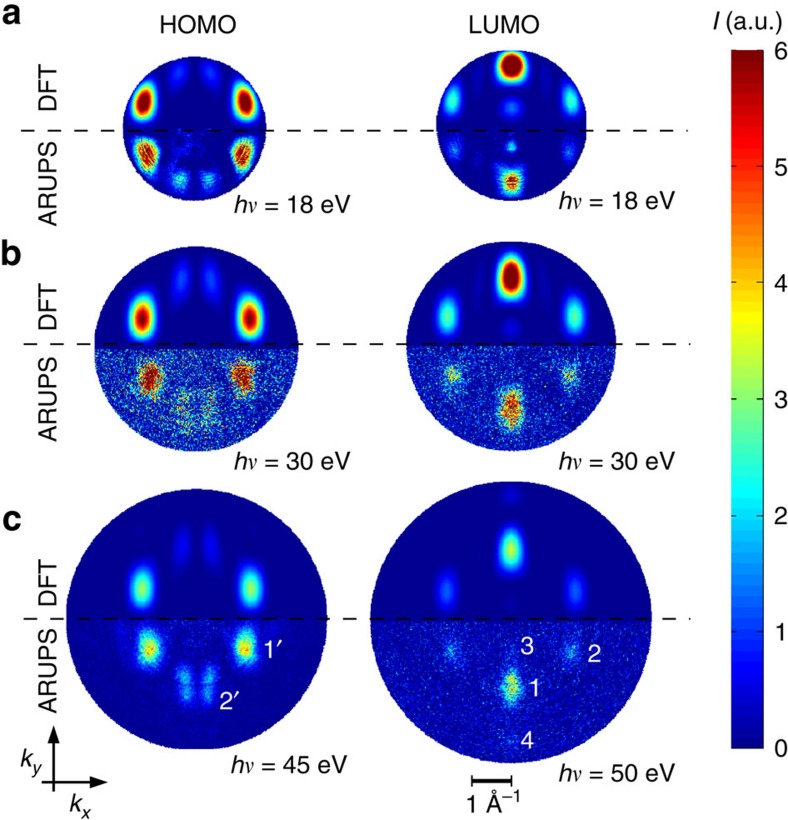
Compilation of experimental and simulated momentum maps of the PTCDA HOMO and LUMO at various photon energies. (**a**–**c**) Momentum maps at photon energies of 18, 30 and 45/50 eV, respectively, where the left (right) column depicts HOMO (LUMO) momentum maps, respectively. In each map, simulated results from a plane wave final state approximation for the free PTCDA molecule (top half of the map) are compared with corresponding ARPES data from the monolayer of PTCDA/Ag(110) (bottom half of the map). Numbers 1–4 in (**c**) refer to the LUMO lobes as defined in Fig. 1, 1' and 2' denote the two main lobes of the HOMO.

Figure 2 compares experimental and theoretical CBE maps of the PTCDA HOMO (left column) and LUMO (right column) for the photon energies 18, 30 and 45/50 eV. Note that the bottom half of each *k*_*x*_*k*_*y*_ map corresponds to experimental data taken on a monolayer of PTCDA/Ag(110) for which it is known that there is charge transfer into the formerly unoccupied LUMO which ends up at a binding energy of 0.8 eV below the Fermi level, while the HOMO has a binding energy of 1.9 eV (refs 8, 17, 26). The upper halves display theoretical results based on hemispherical cuts through the 3D momentum space function 
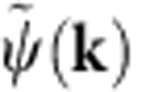
 of the free PTCDA HOMO and LUMO as obtained from DFT for corresponding kinetic energies. Two things must be noted when looking at these *k*_*x*_*k*_*y*_ momentum maps. First, the agreement between theory and experiment is remarkable for all three photon energies. Note that we varied the photon energies in 1–2 eV steps between 15 and 50 eV, the corresponding full set of data is shown in the Supplementary Figs 3 and 4. The overall good agreement between theory and experiment proves that the plane wave final state approximation captures well the angular dependence of the photocurrent for all photon energies in this range. In particular, the lobes already introduced in Fig. 1b appear in, both, experimental and theoretical maps. At *hν*=18 and 30 eV only lobes 1, 2 and 3 are visible, while at 50 eV also lobe 4 has appeared. These features at larger (*k*_*x*_, *k*_*y*_) positions contain information about larger spatial frequencies, thus will enhance the resolution in reconstructed real-space images.

### Photon energy dependence

In the following, we focus on how the photoemission intensity varies with photon energy. Closer inspection of the experimental data suggests that the intensity of any particular lobe exhibits a characteristic behaviour with a maximum of intensity at photon energies around 22 eV. However, for a reliably quantitative determination of the photoemission intensity, care must be taken with the measurement of the photon flux for the different photon energies. This can be done by using absolutely calibrated semiconductor photodiodes as provided by the Physikalisch-Technische Bundesanstalt (PTB)^29^, the German national metrology institute. However, even with such photodiodes the measurement will be erroneous, if the monochromatized synchrotron radiation at the preset photon energy contains contributions from higher grating orders and stray light from the monochromator. Although these effects typically only have a minor influence on photoelectron spectra since the electron analyser is energy-resolving, the signal from the photodiode (and any other photoemissive detector) strongly responds to all spectral contributions. Therefore, it is essential to use a well-characterized synchrotron radiation beamline where any false-light contributions are suppressed as much as possible. For most synchrotron beamlines, this aspect is usually disregarded because they are optimized for maximum photon flux and/or spectral resolution. The experiments reported here were carried out at the Metrology Light Source (MLS) at the PTB. The absolute photon flux was monitored with relative uncertainties from 5 to 10% which comprise a 2–4% contribution from the photon yield calibration (Supplementary Fig. 2), but which are dominated by contributions from temporal variations. More details can be found in the Methods section.

**Figure 3:**
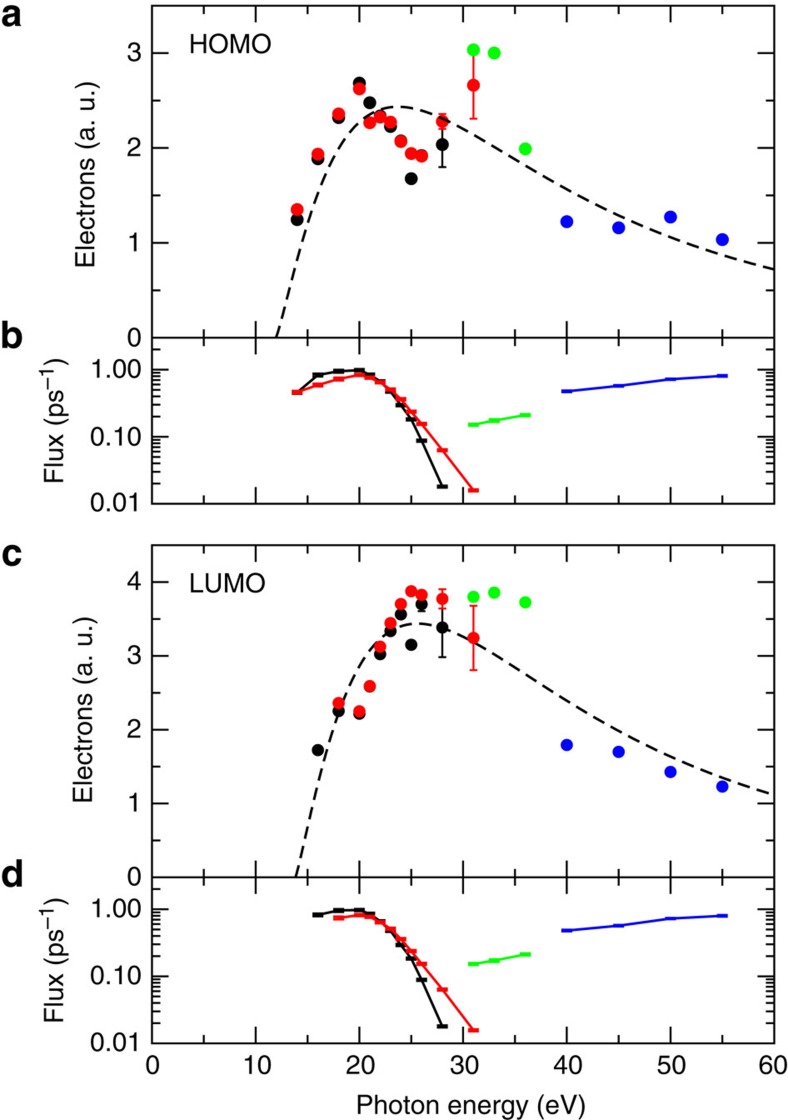
Calibrated photoemission intensities as a function of photon energy. (**a**) Normalized photoemission intensities of the main HOMO lobe marked as 1' in Fig. 2 as a function of photon energy. Symbols correspond to experimental data, the dashed line is a fit within the plane wave final state approximation. (**c**) Same as (**a**) for the LUMO main lobe marked as 1 in Fig. 2. (**b** and **d**) Calibrated photon fluxes for each measurement point. The colours refer to different undulator, monochromator and filter settings as detailed in the Methods section. The error bars represent the standard deviation of the count rate from Poisson statistics.

Based on such a careful calibration of the photon flux, we are able to extract the photoemission intensity of the major lobes of the HOMO and LUMO maps as a function of photon energy. The results are depicted in Fig. 3, while the full sets are presented in the Supplementary Figs. 3 and 4. Details on how the intensities have been extracted are outlined in the Methods section. Inspection of the data points reveals that both, HOMO and LUMO data, essentially show the same photon energy dependence. Starting from the lowest photon energies, the intensity increases and peaks at roughly 20 and 25 eV for the HOMO and LUMO, respectively, then it slightly decreases before a second maximum appears around 33 eV. For larger photon energies, we observe a monotonic decrease of the intensity.

### Plane wave final state prediction

To interpret the observed photon energy dependence, we compare it to the prediction of the plane wave final state approximation. To this end, we convert the measured photoemission intensities *I* to a wave function in momentum space 
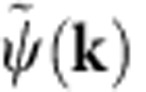
 via the relation


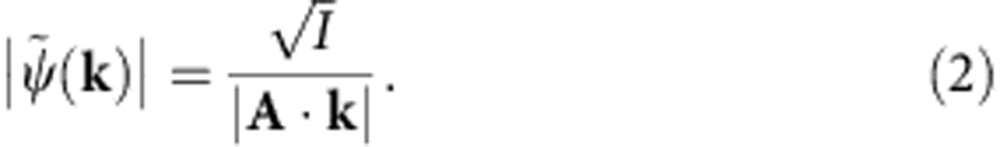


**Figure 4:**
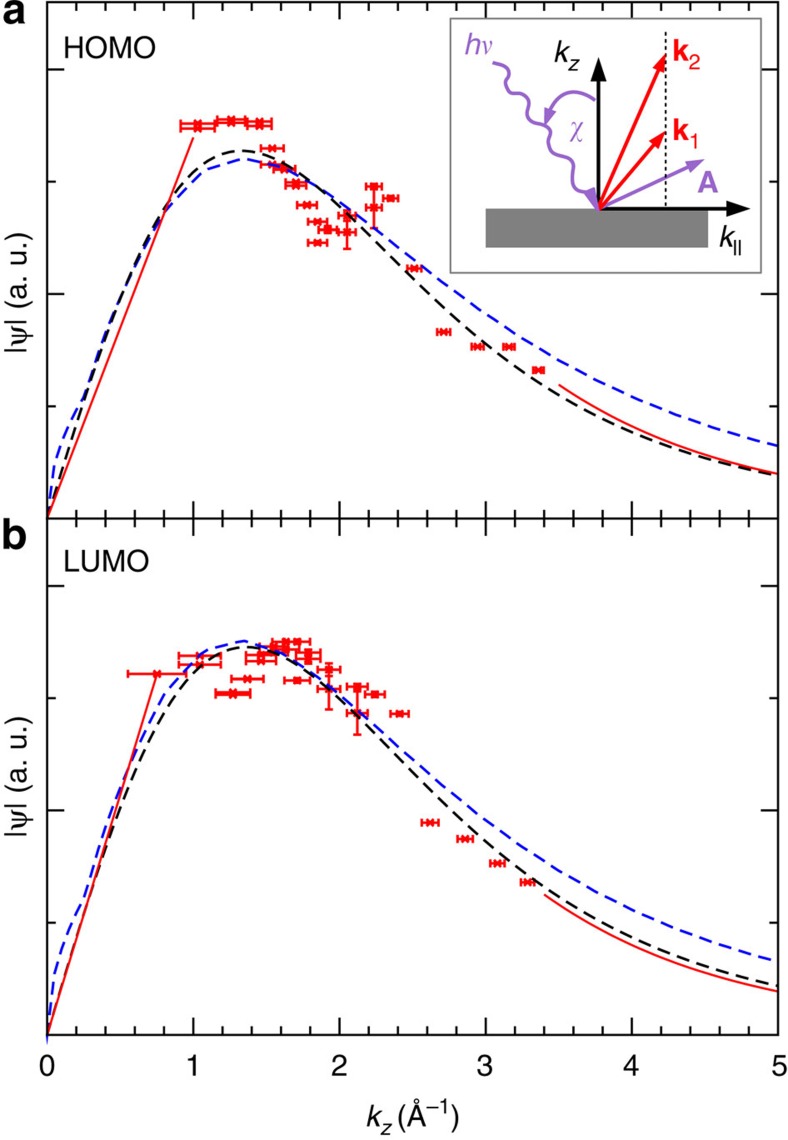
Perpendicular component momentum space representation of HOMO and LUMO. The red symbols show experimental data points for the HOMO (**a**) and LUMO (**b**) as converted according to equation 2, the red lines are data extrapolations as explained in the text. The black dashed lines show fits according to equation 3. The blue dashed lines show the *k*_*z*_-dependence of the DFT HOMO and LUMO orbitals in momentum space. The vertical error bars represent the s.d. of the count rate from Poisson statistics, while the horizontal error bars are standard uncertainties arising from uncertainties in the parallel momentum measurement Δ*k*_||_=±0.1 Å^−1^. The inset in (**a**) is a schematic representation of the experimental geometry as detailed in the text.

Here, the factor **A**·**k** arises from the momentum operator in the transition matrix element, which can be factored out when assuming a plane wave final state. In our experimental setup (see inset of Fig. 4a), using in-plane polarized light at an angle of incidence *χ*=40°, the scalar product **A**·**k** varies with photon energy for two reasons. First, the angle between the polarization vector, which is fixed for all measurements, and the emission direction changes for a fixed *k*_||_ value, and second, also the length of the final **k** increases when varying the photon energy. Moreover, we also convert the photon energy to the perpendicular momentum component *k*_*z*_ by using equation 1 and the Pythagorean 
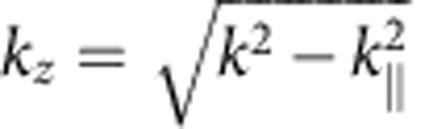
. This leads to the data points shown in Fig. 4. Note that the vertical error flags again represent the s.d. of the count rate from Poisson statistics, while the horizontal error bars are due to an uncertainty in the determination of the parallel momentum component Δ*k*_||_=±0.1 Å^−1^. The two maxima discussed in the *I* versus *hν* plot (Fig. 3) now appear at *k*_*z*_≈1.3 and 2.3 Å^−1^ in this representation, although the height of the higher lying maximum is reduced due to the |**A**·**k**| in the denominator. We can now compare the experimental data with the *k*_*z*_-dependence of the Fourier transforms of the HOMO and LUMO as calculated by DFT. These are shown as blue dashed lines in Fig. 4. Overall, the plane wave final state prediction for a free PTCDA molecule represents the experimental data points surprisingly well. The position of the major maximum and the tailing off at large *k*_*z*_, respectively photon energies, is in reasonable agreement. However, the experimental data appears to exhibit a weak but significant modulation on top of the plane wave final state expectation.

Before discussing possible sources of this additional structure, we further illuminate the plane wave final state prediction which will help us to explain the physical origin of the major *k*_*z*_≈1.3 Å^−1^ maximum. To this end, we compute the analytic form of the Fourier transform of an atomic *p*_*z*_ orbital, 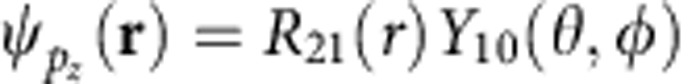, where *R*_21_ is the radial wave function and *Y*_10_ is the spherical harmonic, leading to


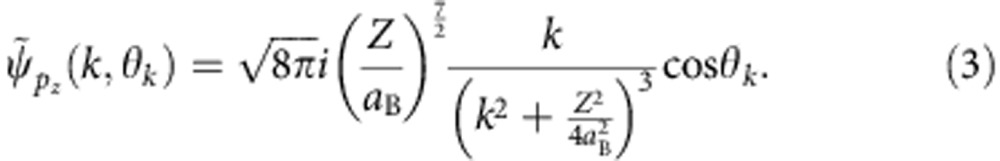


Here, *k* and *θ*_*k*_ are the length and the polar angle of the wave vector **k**, and *Z* and *a*_B_ are the nuclear charge and the Bohr radius, respectively. For normal emission, *θ*_*k*_=0, the function rises as 
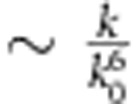
 for small *k*, peaks at 
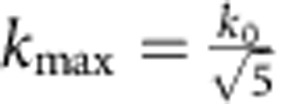
, and tails off as 
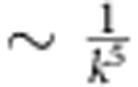
 for *k*→∞, where we have abbreviated 
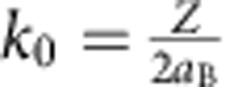
. Thus, the value of *k*_0_ is inversely proportional to the *z*-extension of the orbital which is 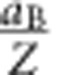. Fitting equation 3 to the experimental data leads the black dashed line in Fig. 4. As best fit parameters, we obtain *k*_0_ values of 2.85 and 2.97 Å^−1^ for the HOMO and LUMO, respectively, resulting in the main peaks at 
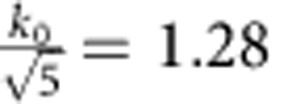
 and 1.33 Å^−1^. In particular the former value for the HOMO is in agreement with the DFT result (blue dashed line), but also the 4% larger value for the LUMO, indicating an ≈4% *z*-reduction of the LUMO orbital in real space, agrees within experimental uncertainties, with the DFT wave function. Note that the fitted curves (black dashed lines), when converted into *I* versus *hν* plots by reversing the procedure outlined above, lead to the dashed lines displayed in Fig. 3. Clearly, the additional modulation seen in the data, for instance, the peak/shoulder at *k*_*z*_=2.3 Å^−1^ (the 33 eV maximum of Fig. 3) cannot be explained when assuming a plane wave for the final state. We also note that by taking the square root of the data, which is necessary to reconstruct the orbital in real space, this additional structure seen in the raw data (Fig. 3) is diminished and appears only as a rather weak modulation of the plane wave final state expectation.

### Three-dimensional orbital images

**Figure 5:**
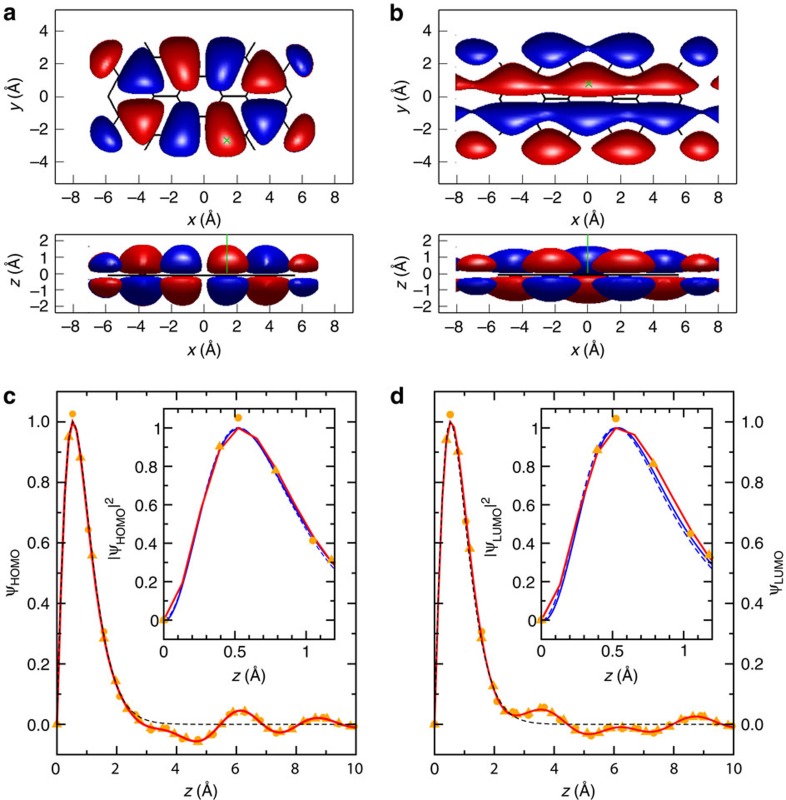
Reconstructed HOMO and LUMO orbitals of PTCDA in a real space representation. (**a**) 3D images of the HOMO in top view and side view respectively. (**b**) The same for the LUMO. Red (blue) regions indicate isosurfaces corresponding to a constant positive (negative) value of the wave function. (**c** and **d**) Vertical line scans through the HOMO and LUMO wave functions 
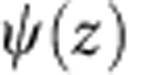
, respectively, at the positions indicated in green. The red solid lines and the orange symbols are reconstructed from the raw measurement data extrapolated up to *k*_max_=24 Å^−1^ (red line), 8 Å^−1^ (orange triangles) or 6 Å^−1^ (orange circles), respectively, while the black dashed lines are based on a plane wave fit when performing the reconstruction. The insets in (**c**) and (**d**) show zoomed regions around the maximum electron density 
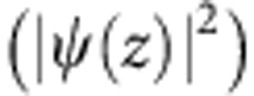
. Here, the solid (dashed) blue lines are vertical line scans through the DFT HOMO and LUMO orbitals of a free-standing (adsorbed) PTCDA molecule which are compared with the reconstructed orbitals.

To obtain a more quantitative picture of the three-dimensional charge distribution of HOMO and LUMO orbitals in real space, we perform a full 3D reconstruction of the orbital from the ARPES data (Fig. 5). Here, we combine the (*k*_*x*_, *k*_*y*_) momentum map including the numerically recovered phase^17^ with the *k*_*z*_-dependence discussed above and compute the 3D orbital in real space from an inverse Fourier transform. To this end, we make use of the measured data which contains the crucial features, namely the maxima and the tailing off towards large *k*_*z*_. However, to allow for an inverse Fourier transform, we must extrapolate the data outside the measurement window to avoid truncation effects. Intensity values for *k*_*z*_
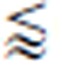
0.8 Å^−1^, corresponding to *hv*
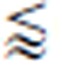
15 eV, are not experimentally accessible as the wave number of the emitted electron (the radius of the sphere in Fig. 1b) falls below the *k*_||_ component of the principal HOMO and LUMO lobes 1' and 1. Nevertheless, we can expect 
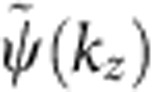
 to approach zero for *k*_*z*_=0 due to the nodal plane of the *π*-orbital. For simplicity, we choose here a straight line as indicated by the red line in Fig. 4. Intensity values beyond *k*_*z*_
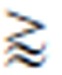
3.4 Å^−1^, corresponding to *hv*
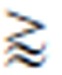
55 eV, are in principle approachable via experiment. They are, however, problematic as, apart from film damage, the increase in mean free path length of substrate photoelectrons combined with the overall decrease of the molecular signal leads to a poor signal-to-background ratio with increasing photon energy. Since scattering effects are expected to diminish for final states well above the vacuum level, extrapolating the data by a ∼1/*k*^5^ tail (red line in Fig. 4), as expected for a plane wave final state, is a reasonable assumption. Finally, we impose 
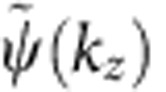
 to be anti-symmetric with respect to *k*_*z*_, leading to a charge distribution that is symmetric about the molecular plane, an assumption which is confirmed by DFT calculations of the PTCDA/Ag(110) interface.

The reconstructed HOMO and LUMO orbitals resulting from this procedure are shown in Fig. 5, panels (a) and (b), respectively. Note that the top views are equivalent in shape to the two-dimensional reconstructions presented earlier^17^ and are in excellent agreement with the DFT orbitals. Also, the side views reveal an overall good agreement with the vertical charge extension resulting from DFT. To analyse the vertical extension in more detail, panels (c) and (d) depict line scans of the reconstructed orbitals along the *z* direction at positions indicated by the green dashed lines in (a) and (b). The red lines result from an inverse Fourier transform of the data which has been extrapolated as described above. It is important to note that the choice of *k*_max_=24 Å^−1^ determines the spatial resolution in *z* direction Δ*z*=*π*/*k*_max_, however, does not affect the overall shape of the orbital in real space, that is its vertical extension and width. This is demonstrated by the orange symbols resulting from inverse Fourier transforms with significantly smaller *k*_max_ values of 6 Å^−1^ (circles) and 8 Å^−1^ (triangles), respectively. Note that with *k*_max_ values significantly smaller than 6 Å^−1^ or without any extrapolation, the spatial resolution will get unfavourable and truncation effects start to appear due to the sudden step in the data. The HOMO and LUMO wave functions peak at *z*=0.55 and 0.53 Å, respectively, which can be seen more clearly in the insets. Taking the plane wave fit for the reconstruction (the dashed black lines in Figs 3 and 4), we obtain the black dashed lines shown in Fig. 5c,d. It agrees with the former in terms of the position and width of the major maximum but tails off smoothly towards zero for large *z* where the former exhibits oscillations owing to the additional structure in the data. However, these oscillations at large *z* will not show up in typical isosurface representations of orbitals. This can be seen in Fig. 5a,b where we show an isosurface of 17% of the maximum which contains about 90% of the orbitals' charge density. When comparing the experimental findings with the orbital extension predicted from DFT, we see that the *z*-extensions of the reconstructed HOMO and LUMO wave functions are only marginally different from the theoretical results (peak at *z*=0.54 Å for both, the HOMO and LUMO), irrespective of whether the free-standing orbitals (blue solid line) or the orbital of the adsorbed PTCDA/Ag(110) monolayer (blue dashed line) are considered.

## Discussion

Our careful analysis of the photon energy-dependent measurements indicate that indeed a reconstruction of molecular orbitals in three dimensions from photoemission intensity data seems possible although there is evidence in the raw data for features attributable to final state effects beyond the plane wave approximation. For instance, the feature around *k*_*z*_=2.3 Å^−1^ cannot be explained by a modification of the initial state wave function arising from molecule–substrate interactions as our DFT calculations for the PTCDA/Ag(110) interface have shown. Thus, if chemical effects upon adsorption can be ruled out, we must look for alternative explanations.

We suggest that these secondary modulations may result from the scattering of the outgoing electrons. Indeed, one might be tempted to identify this modulation with either a photoelectron diffraction or alternatively a final state resonance effect. Although the modulation period seems too short to be attributed to scattering from the outermost substrate layer, the global potential defined by the dimensions of the molecule may well give rise to final state resonances (so-called shape resonances) which appear at certain kinetic energies of the outgoing photoelectron. This phenomenon has been well-studied in near-edge X-ray absorption fine structure (NEXAFS). Shape resonances have in fact been used to determine interatomic bond lengths^30^. For our large molecule, shape resonances connected with the lateral dimensions are expected to occur at very-low photon energies and corresponding electron kinetic energies below our measurement region. Interestingly, however, a resonance at *k*_*z*_≈2.3 Å^−1^ would correspond to a vertical distance of 
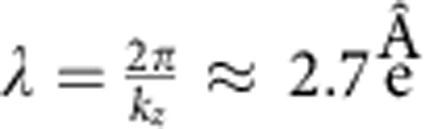
, which approximately corresponds to the adsorption height of PTCDA on Ag(110) (2.56 Å)^26^,^31^,^32^.

Although the attempts to understand the observed weak additional modulation in the photoelectron intensity remain speculative at this stage, the energy dependence is dominated by the form expected from the plane wave final state. Furthermore, because it is the square root of the intensity which enters the reconstruction, the weak modulation is further damped (cf. Figs 3 and 4). As a result, the reconstructed orbitals are rather insensitive to this modulation.

At this point, the question to ask is why does the very simple plane wave approximation perform so well at all^27^? Of course, there are technical aspects to this answer. As pointed out above, imaging in the appropriate region of *k*-space, choosing an experimental geometry for which the polarization term |**A**·**k**| is fairly constant and as large as possible, and carrying out a careful normalization of the photoelectron intensity are all essential prerequisites for a successful orbital reconstruction. But beyond these technical issues, still the questions remains why the final state can be described well as a plane wave, for the systems which we have investigated so far. We believe that this has to do with the comparably large size of the molecules under study (for instance, in relation to diatomic molecules), in conjunction with the fact that the electrons in the initial state are delocalized over the entire molecule. Thereby, the final state may be viewed as a superposition of partial waves emitted from all atomic centres being part of a given molecular orbital which, according to Huygens' principle, add up to a plane wave, provided that the individual waves are in phase. This requirement is fulfilled if all atomic potential wells are of similar depth. As a consequence, the angular dependence of the photocurrent at any particular final kinetic energy is well captured by the Fourier transform of the initial state.

In a sense, it is reassuring that in this work we have observed deviations from the prediction of the plane wave final state, because this allows us for the first time to quantify the upper limit of photoelectron diffraction and/or final state scattering effects in the systems under study. In our view, this puts the application of the orbital tomography approach on molecular adsorbate systems on a much firmer ground. Finally, our work also suggests how to advance the orbital tomography approach in future studies. Addressing theory, it will be interesting to go beyond the plane wave approximation and take into account resonances in the final state. This can, for instance, be achieved by incorporating the surface potential by solving the Lippmann–Schwinger equation. Concerning future experiments, as well as extending the photon energy range, it will be illuminating to perform photon energy-dependent ARPES experiments for different organic monolayers with varying interaction strengths and adsorption heights. One goal in this context is searching for adsorbate systems with strong orbital modifications that could be imaged with the 3D reconstruction. Another direction will be the investigation of final state resonances, and in particular, correlations between their energy position and the adsorption height.

## Methods

### Sample preparation

For straightforward orbital reconstruction it is advantageous to investigate an adsorbate system where all molecules are oriented in the same direction. Otherwise the angle-resolved photoemission data would be a superposition of the different molecular orientations and the Fourier transformation would lead to a superposition of differently oriented orbitals. For our proof-of-principle experiment we chose PTCDA adsorbed on the Ag(110) surface. Depending on the preparation there exist two monolayer phases of PTCDA/Ag(110): brickwall and herringbone. In the brickwall structure that was studied here all molecules are oriented with their long axis parallel to the [001] direction of Ag(110)^12^. The sample was prepared and investigated under ultra high vacuum conditions. Several cycles of sputtering with Ar^+^ ions and subsequent annealing were performed to clean the surface of the Ag(110) crystal. A submonolayer of PTCDA molecules was deposited at room temperature on the surface using a home-built Knudsen cell. The structure of the layer was checked with low-energy electron diffraction to ensure that the investigated structure is indeed the brickwall and not the herringbone monolayer.

### Analyser

The ARPES experiments were performed with a toroidal analyser^33^. The advantage of this type of analyser is that it allows simultaneous collection of photoelectrons emitted with polar angles ranging from −85° to +85° in the plane of incidence of the synchrotron light and in an energy dispersion range of ∼1 eV. After passing the dispersive element the photoelectrons form an arc on the micro-channel plate detector with polar angle *θ* and kinetic energy *E*_kin_ mapped in azimuthal and radial directions, respectively. The image is recorded with a charged-coupled device camera and the arc is divided into sectors, leading to an intensity distribution *I*_1_(*θ*,*E*_kin_,*hν*). To change the plane of incidence with respect to the sample, the latter can be rotated around the surface normal by azimuthal angles *φ*. Hence it is possible to map the nearly complete positive half-sphere of momentum space, yielding *I*_1_(*φ*,*θ*,*E*_kin_,*hν*).

### Synchrotron radiation beamline

Experiments were carried out with synchrotron radiation at the beamline IDB (Insertion Device Beamline) at the MLS of PTB in Berlin^29^. At this beamline, false-light contributions are suppressed below 10^−2^ by using suitable order-sorting filters. To obtain synchrotron light in the desired energy range from 14 to 55 eV, we employ four different undulator/monochromator/filter configurations: (1) the first undulator harmonic with normal incidence onto the grating monochromator without a filter (14–28 eV, black in Fig. 3 and Supplementary Fig. 2), (2) the third harmonic with normal incidence without a filter (14–31 eV, red), (3) the third harmonic with grazing incidence in combination with a Mg filter (31–36 eV, green) and (4) the sixth harmonic with grazing incidence with a Al filter (40–55 eV, blue). During all measurements, the photon flux is continuously monitored by a reference detector, namely the photoemission from a beamline mirror surface. This yields the time-dependent mirror current *i*(*t*). This mirror current *i*(*t*) was regularly measured against an absolutely calibrated semiconductor photodiode as our detector standard. This comparison yields the energy-dependent photon yield curve *η*(*hν*), measured in units of photons per seconds and nanoampere mirror current (Supplementary Fig. 2). The combination of proper false-light suppression, permanent mirror current monitoring and regular comparison to an absolutely calibrated photodiode allows us to determine the flux of photons with the specified energy for each measurement with relative uncertainties from 5 to 10% which comprise a 2–4% contribution from the photon yield calibration (Supplementary Fig. 2), but which are dominated by contributions from temporal variations.

### Measurement

For CBE maps, the sample is rotated around the surface normal, with the azimuthal angle *φ* changing in 1° steps, while both the photon energy and kinetic energy of the collected photoelectrons are kept constant. In this way a half-spherical cut through *k*-space is mapped see Fig. 2. Assuming that the intensity of the full map *I*(*k*_*x*_,*k*_*y*_) scales with the intensity of its main lobes, the intensity versus photon energy curves of Fig. 3 in the main text are based on measurements of a 10° azimuthal-sector around the main lobes of HOMO and LUMO, respectively. At selected photon energies 180° scans were performed. Their analysis corroborates the above assumption.

### Treatment of raw data

For further analysis we only consider photoelectrons emitted in forward direction (*θ*<0). For each CBE measurement we obtain an intensity distribution *I*_1_(*φ*,*θ*,*E*_kin_,*hν*). In the first step we subtract the dark count rate which increases with the radius within the arc on the detector. This yields *I*_2_(*φ*,*θ*,*E*_kin_,*hν*). Rare occasional glitches in the raw data are eliminated by averaging adjacent data points. *I*_2_(*φ*,*θ*,*E*_kin_,*hν*) still contains a diffuse background that increases towards the Γ-point (*θ*=0), see Supplementary Fig. 1. To eliminate this background we apply the following procedure. The average 〈*I*_2_(*φ*,*θ*,*E*_kin_,*hν*)〉_*φ*_ is calculated and fitted in the range −85°<*θ* <+80.5° and −4.5°<*θ* <0° with a parabola *p*(*θ*,*E*_kin_,*hν*). The background corrected intensity *I*_3_(*φ*,*θ*,*E*_kin_,*hν*) is calculated by *I*_3_(*φ*,*θ*,*E*_kin_,*hν*)=*I*_2_(*φ*,*θ*,*E*_kin_,*hν*)−*p*(*θ*,*E*_kin_,*hν*). Next the (*φ*,*θ*)-dependence is converted into parallel momentum components using the equations 
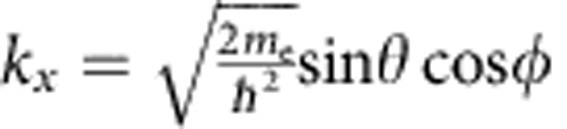
 and 
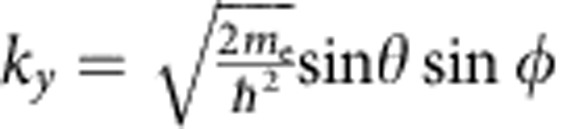
. This yields CBE maps *I*(*k*_*x*_,*k*_*y*_,*E*_kin_,*hν*) as depicted in the Supplementary Figs 3 and 4. In the CBE maps *I*(*k*_*x*_,*k*_*y*_,*E*_kin_,*hν*) the intensity is averaged over the (*k*_*x*_,*k*_*y*_)-region belonging to the main lobe (red box in Supplementary Fig. 4). The final but not yet normalised *I*(*hν*) curve is obtained by selecting for each photon energy the intensity *I*(*E*_kin_^max^,*hν*), where *E*_kin_^max^ is the kinetic energy at which the maximum of the intensity *I*(*E*_kin_,*hν*) is located. For the error bars we use 
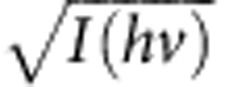
.

### Intensity normalization

As mentioned in the main text, normalizing the intensities *I*(*hν*) by the actual photon flux is a crucial step. For each CBE measurement at a given *hν* the average of the mirror current 〈*i*_m_(*t*)〉_*t*_ is calculated and multiplied by the corresponding photon energy-dependent photon yield *η*(*hν*) (Supplementary Fig. 2). This yields the average photon flux Φ(*hν*)=〈*i*_m_(*t*)〉_*t*_·*η*(*hν*). The normalized intensities *I*_n_(*hν*) are calculated as 
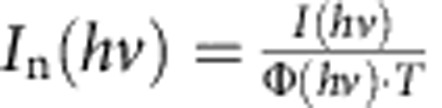
, where *T* is the acquisition time of the camera. The error bars of *I*_n_(*hν*) are calculated from those of *I*(*hν*) and *η*(*hν*). Results are shown in Fig. 3.

### Computational details

All theoretical results presented in this work have been obtained within the framework of DFT. For the computation of the electronic structure of the PTCDA/Ag(110) we employed a repeated slab approach using the VASP code^34^,^35^. The Ag(110) substrate was modelled by six metallic layers with an additional vacuum layer of ≈15 Å. The generalized gradient approximation^36^ is used for exchange-correlation effects, and the projector-augmented waves^37^ approach was used allowing for a relatively low kinetic energy cut-off of ∼400 eV. We use a Monkhorst-Pack 6 × 6 × 1 grid of *k*-points^38^, and a first-order Methfessel-Paxton smearing of 0.05 eV (ref. 39). To avoid spurious electrical fields, a dipole layer is inserted in the vacuum region^40^. To circumvent issues concerning van-der-Waals interactions which are ill-described in standard GGA functionals^41^,^42^, we employ the empirical correction scheme according to Grimme^43^. During the geometry optimization of the internal atomic positions we fixed the height of the carbon atoms of the molecule at the experimentally determined values^26^,^32^ and allowed for a full relaxation of the other atoms of the molecule as well as the two topmost layers of the metal substrate. Note that as a cross-check, we have also performed a full relaxation using the vdW-surf method by Ruiz *et al*.^44^. The resulting adsorption height agrees with the experimental one where the differences are below ±0.1 Å. Consequently, the so-obtained partial charge densities of the HOMO and LUMO are virtually indistinguishable from the ones obtained by fixing the carbon atoms at the experimental heights.

To calculate momentum maps of the free PTCDA molecule, we utilize the plane wave code ABINIT^45^. Here the all-electron potentials were replaced by extended norm-conserving, highly transferable Troullier-Martins pseudo potentials^46^ using a plane wave cut-off of 50 Ryd. We employ a super cell approach with a box size of 50 × 50 × 25 Bohr^3^ and Γ point sampling of the Brillouin zone. The geometry of the free molecule is optimized by using GGA^36^ for exchange-correlation effects.

## Additional information

**How to cite this article:** Weiß, S. *et al*. Exploring three-dimensional orbital imaging with energy dependent photoemission tomography. *Nat. Commun.* 6:8287 doi: 10.1038/ncomms9287 (2015).

## Supplementary Material

Supplementary Information Supplementary Figures 1-4
