# Supplementary material for: Exploring three-dimensional orbital imaging with energy-dependent photoemission tomography
Source: Nat Commun. 2015 Oct 5;6:8287. doi: 10.1038/ncomms9287 (PMC4600719; doi:10.1038/ncomms9287)
Supplement: Supplementary Information — Supplementary Figures 1-4 [file ncomms9287-s1.pdf]

SUPPLEMENTARY FIGURES

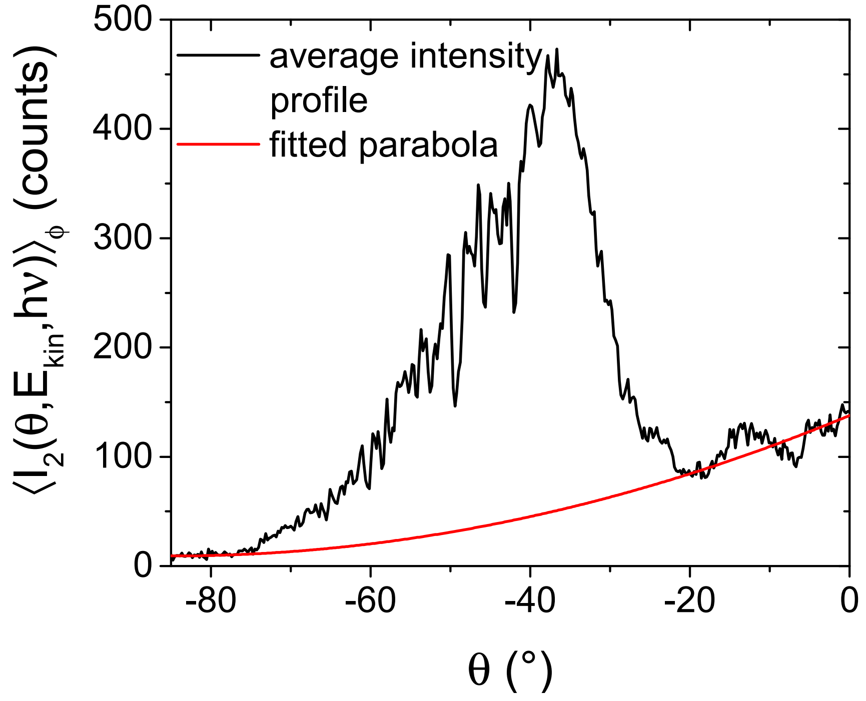

Supplementary Figure 1: Illustration of the dark count rate background subtraction. A parabola $p(\theta, E_{\rm kin}, h\nu$ (red) is fitted to the average profile $\langle I_2(\phi, \theta, E_{\rm kin}, h\nu)\rangle_\phi$ (black) for constant $E_{\rm kin}$ and $h\nu$ to eliminate diffuse background around Γ-point.

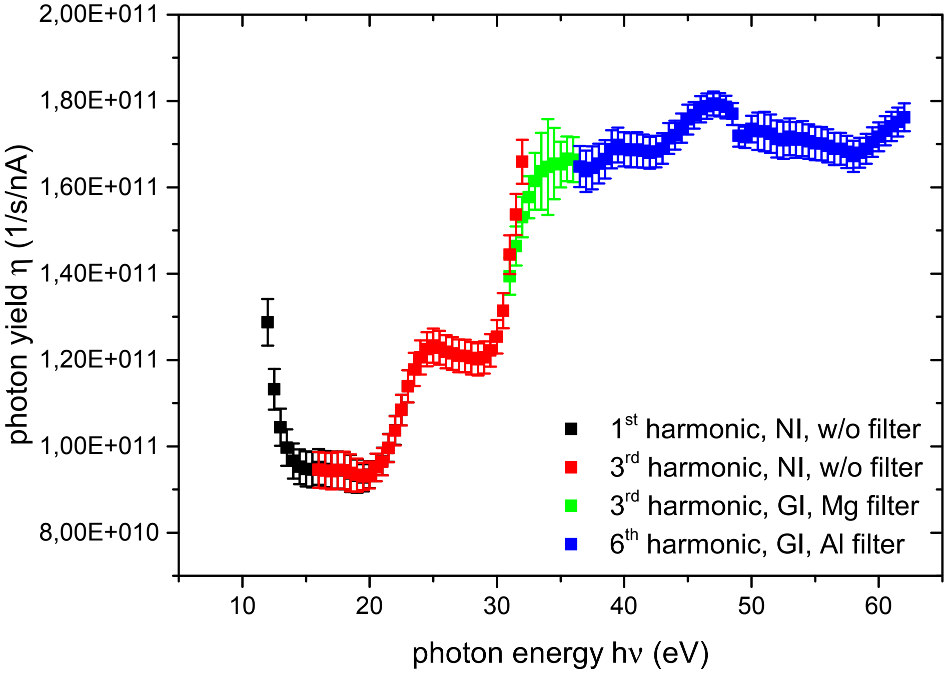

Supplementary Figure 2: Energy dependent photon yield curve. The photon yield $\eta(h\nu)$ is used to calculate the photon flux $\Phi(h\nu) = \eta(h\nu)i_{\mathrm{m}}$ from the mirror current i_{m} during a CBE measurement. This curve is the result of calibration measurements, where the photon-flux and the mirror current are measured simultaneously. Colors as in Fig. 3 of the main text, NI and GI denotes normal normal and grazing incidence, respectively.

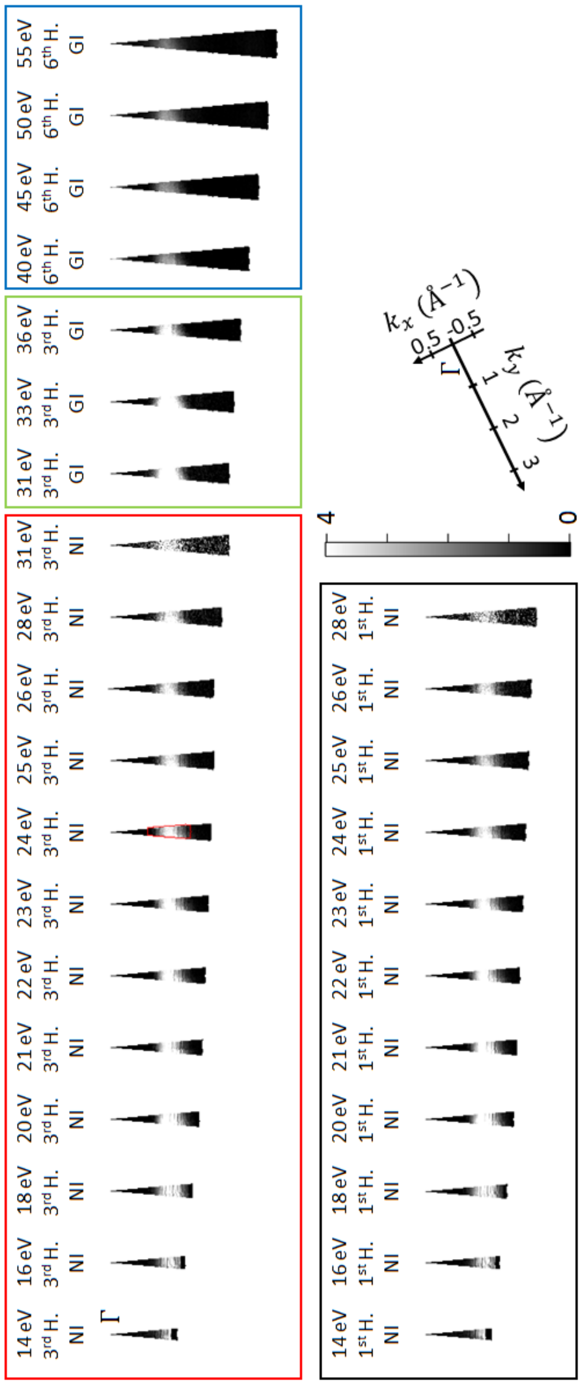

Supplementary Figure 3: Compilation of 10°-sectors of constant binding energy maps at the HOMO energy for various photon energies. These 10°-sectors of the HOMO-CBE-maps were measured to gain intensity vs. photon energy information. This image shows $\frac{I(\phi,\theta,E_{\mathrm{kin}},h\nu)}{\Phi(h\nu)\cdot T}$, hence the normalized CBE maps. The red box at the 24 eV map indicates the (k_x, k_y)-region where the intensities were averaged to gain $I(E_{\mathrm{kin}}, h\nu)$. The labels specify the corresponding photon energy, beamline harmonics and the grating.

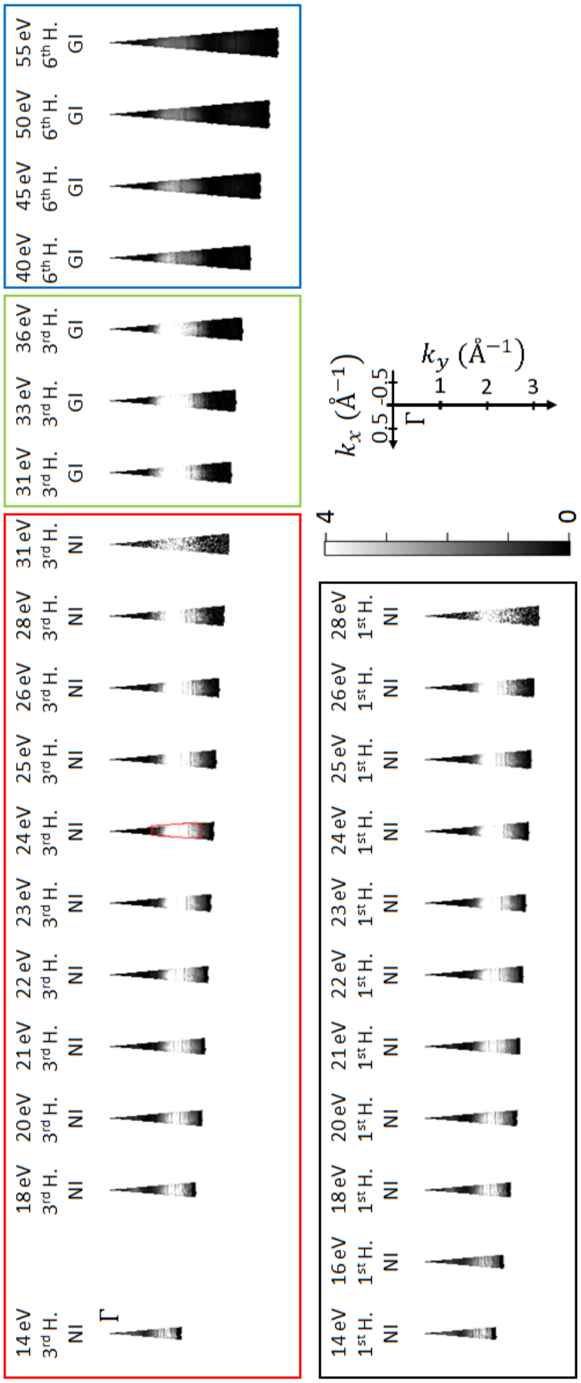

Supplementary Figure 4: Compilation of 10°-sectors of constant binding energy maps at the LUMO energy for various photon energies. These 10°-sectors of the LUMO-CBE-maps were measured to gain intensity vs. photon energy information. This image shows $\frac{I(\phi,\theta,E_{\text{kin}},h\nu)}{\Phi(h\nu)\cdot T}$, hence the normalized CBE maps. The red box at the 24 eV map indicates the (k_x, k_y)-region where the intensities were averaged to gain $I(E_{\text{kin}}, h\nu)$. The labels specify the corresponding photon energy, beamline harmonics and the grating.
